# Supplementary material for: Initial elevations in glutamate and dopamine neurotransmission decline with age, as does exploratory behavior, in LRRK2 G2019S knock-in mice
Source: eLife. 2017 Sep 20;6:e28377. doi: 10.7554/eLife.28377 (PMC5633343; doi:10.7554/eLife.28377)
Supplement: Transparent reporting form [file elife-28377-transrepform.docx]

***eLife’s* transparent reporting form**

We encourage authors to provide detailed information *within their submission* to facilitate the interpretation and replication of experiments. If you have any questions, please contact us: [editorial@elifesciences.org](mailto:editorial@elifesciences.org).

**Sample-size estimation**

- You should state whether an appropriate sample size was computed when the study was being designed
- You should state the statistical method of sample size computation and any required assumptions
- If no explicit power analysis was used, you should describe how you decided what sample (replicate) size (number) to use

Please outline where this information can be found within the submission (e.g., page numbers or figure legends), or explain why this information doesn’t apply to your submission:

For unbiased assessment of animal weight, we included data from all animals that underwent surgery and separately weighed a large portion of our colony at one time point. Standard error is extremely low in all age bins, which have a minimum of seven observations but are for the most part much higher. For behavioral analyses of exploratory activity, we have found through many years of published data that cohorts of 15 animals per genotype are required; however, mouse behavioral assessment suffers from many uncontrolled variables and we have increased such a sample size comparing young and aged mice (33-72 mice). For electrophysiological and voltametric recording in the striatum of acute brain slices, a minimum observation of 12-20 cells / slices from a minimum of 4 independent animals per group is common, to supersede chance observations from endogenous activity. Our data sets emerge from 5-22 animals). For drug before and after drug effects and within-cell measures such as ratios of AMPA to NMDA, a difference is internally controlled and therefore we are comfortable with lower animal replicate numbers. While we attempt to ensure that we have more than enough observations to reach predictive power, we are constrained by biology, ageing and the practical limits of animal work. That said, we claim no absolutes, discuss only probability and find our group sizes to be at least double that of other similar studies, in most cases. Similarly, for histological analyses, we sample from a minimum of four independent animals and several cells or image fields, which are themselves also sampled to mean values.

**Replicates**

- You should report how often each experiment was performed
- You should include a definition of biological versus technical replication
- The data obtained should be provided and sufficient information should be provided to indicate the number of independent biological and/or technical replicates
- If you encountered any outliers, you should describe how these were handled
- Criteria for exclusion/inclusion of data should be clearly stated
- High-throughput sequence data should be uploaded before submission, with a private link for reviewers provided (these are available from both GEO and ArrayExpress)

Please outline where this information can be found within the submission (e.g., page numbers or figure legends), or explain why this information doesn’t apply to your submission:

•	You should report how often each experiment was performed

*For staining and immunofluorescence sections were processed once. For every experiment the n is included as are details of ANOVA F values in the text or entire readout tables in figure supplements*

•	You should include a definition of biological versus technical replication

*There is no technical replication, all measures are single biological observations excepting evoked currents for NMDA:AMPA ratios where each current is analyzed from an average of three sweeps.*

•	The data obtained should be provided and sufficient information should be provided to indicate the number of independent biological and/or technical replicates

*Information is included throughout*

•	If you encountered any outliers, you should describe how these were handled

*Any data outliers are included in the data sets. Quality control within the electrophysiology is as detailed in the methods.*

•	Criteria for exclusion/inclusion of data should be clearly stated

*Quality control is as detailed in the methods.*

•	High-throughput sequence data should be uploaded before submission, with a private link for reviewers provided (these are available from both GEO and ArrayExpress)

*N/A*

**Statistical reporting**

- Statistical analysis methods should be described and justified
- Raw data should be presented in figures whenever informative to do so (typically when N per group is less than 10)
- For each experiment, you should identify the statistical tests used, exact values of N, definitions of center, methods of multiple test correction, and dispersion and precision measures (e.g., mean, median, SD, SEM, confidence intervals; and, for the major substantive results, a measure of effect size (e.g., Pearson's r, Cohen's d)
- Report exact p-values wherever possible alongside the summary statistics and 95% confidence intervals. These should be reported for all key questions and not only when the p-value is less than 0.05.

•	Statistical analysis methods should be described and justified

*As detailed in the methods and all statistical reporting is clearly present for each data set and supplemental figures. 2way ANOVA and Holm-Sidak post tests are used throughout, with the exception of a solitary t-test in a figure supplement*

•	Raw data should be presented in figures whenever informative to do so (typically when N per group is less than 10)

*Raw data is present throughout, excepting 2-way RM plots for clarity.*

•	For each experiment, you should identify the statistical tests used, exact values of N, definitions of center, methods of multiple test correction, and dispersion and precision measures (e.g., mean, median, SD, SEM, confidence intervals; and, for the major substantive results, a measure of effect size (e.g., Pearson's r, Cohen's d)

*Sufficient reporting is present throughout in terms of mean, SEM and n.*

•	Report exact p-values wherever possible alongside the summary statistics and 95% confidence intervals. These should be reported for all key questions and not only when the p-value is less than 0.05.

*We feel we have included sufficient reporting throughout in terms of mean, SEM, ANOVA and all p values.*

Please outline where this information can be found within the submission (e.g., page numbers or figure legends), or explain why this information doesn’t apply to your submission:

(For large datasets, or papers with a very large number of statistical tests, you may upload a single table file with tests, Ns, etc., with reference to page numbers in the manuscript.)

**Additional data files (“source data”)**

- We encourage you to upload relevant additional data files, such as numerical data that are represented as a graph in a figure, or as a summary table
- Where provided, these should be in the most useful format, and they can be uploaded as “Source data” files linked to a main figure or table
- Include model definition files including the full list of parameters used
- Include code used for data analysis (e.g., R, MatLab)
- Avoid stating that data files are “available upon request”

Please indicate the figures or tables for which source data files have been provided:

None as yet, but we feel the editors will be satisfied with the level of reporting in the document
